# SecY-SecA fusion protein retains the ability to mediate protein transport

**DOI:** 10.1371/journal.pone.0183434

**Published:** 2017-08-18

**Authors:** Yasunori Sugano, Arata Furukawa, Osamu Nureki, Yoshiki Tanaka, Tomoya Tsukazaki

**Affiliations:** 1 Graduate School of Biological Sciences, Nara Institute of Science and Technology, Ikoma, Nara, Japan; 2 Department of Biological Sciences, Graduate School of Science, The University of Tokyo, Tokyo, Japan; University of California - Davis, UNITED STATES

## Abstract

In bacteria, the membrane protein complex SecY/E/G and SecA ATPase are essential for protein translocation. About 30% of newly synthesized proteins in the cytosol are targeted to and translocated across the cytoplasmic membrane by the Sec factors. Although a number of single-molecule analyses and structural studies, including the crystal structure of SecYEG complexed with SecA, have been published, the underlying molecular mechanisms and the functional oligomer states remain elusive. In this study, we constructed a fusion protein SecY-SecA, which induces the formation of the SecY-A/SecE/SecG complex (SecYAEG), to enable investigation of the molecular mechanisms by advanced single-molecule analyses. SecYAEG-reconstituted liposomes were found to possess protein translocation activity *in vitro* and form stable intermediates capable of the translocation using a mutant substrate protein. We additionally found that one unit of SecYAEG complex embedded into a nanodisc, using membrane scaffold proteins, interacts strongly with the substrate. The isolated SecYAEG-reconstituted nanodisc is a promising tool for investigation of the molecular mechanisms by which a single unit of Sec machinery mediates protein translocation.

## Introduction

Biological membranes, which separate cellular compartments and cells from the environment, generally prevent free diffusion of ions and small molecules. Dedicated protein-conducting channels mediate protein translocation across the membrane. The Sec translocon, an evolutionally conserved protein-conducting channel, exists in all organisms. In bacteria, about 30% of proteins synthesized in the cytoplasm are transported across the cytoplasmic membrane to the periplasmic space *via* the Sec translocon [1]. This process corresponds to protein transport from the cytoplasm into the endoplasmic reticulum lumen in eukaryotes [2, 3]. The bacterial Sec translocon is a heterotrimeric complex composed of three membrane proteins: SecY, SecE, and SecG. In the resting state, the Sec channel is closed; this state is stabilized by a plug from the periplasmic side as well as covered by a loop of SecG from the cytoplasm side [4, 5]. During protein translocation, the plug and the cover are dislocated, and a membrane-penetrated pathway is formed in the center of the Sec translocon [6]. Secretory proteins that have an N-terminal signal peptide are co-translationally or post-translationally targeted to the membrane and translocated *via* the Sec translocon. In the co-translational process, the ribosome-nascent chain complex directly binds to the Sec translocon, allowing the polypeptides emerging form the ribosome exit tunnel to be transported while maintaining the unfolded state. Single-particle cryo-electron microscopy studies have shown that one unit of Sec translocon forms the protein-conducting channel in this process [7–10]. In the case of post-translational translocation in bacteria, the synthesized secretory proteins in the cytoplasm are maintained in unfolded states by chaperones, such as SecB, and then targeted to the SecYEG. SecA ATPase complexed with SecYEG functions as the essential cytosolic protein translocation motor [11, 12]. Repetitive conformational transitions of SecA coupled with ATP hydrolysis push the substrate into the SecYEG channel. On the periplasmic side, the secondary motor SecDF, driven by proton motive force, supports protein translocation [13, 14]. The crystal structures of the Sec translocon [4, 5, 15–17] suggest that one unit of the SecYEG complex exists stably. The recently reported structural analysis of Sec complex [6] indicates that a single unit of the SecYEG complex and SecA form an intermediate complex with a translocating protein. However, some reports show that the dimeric SecYEG complex represents the functional unit [18–20]. The functional oligomeric states of SecA are still controversial [1, 6, 21]. According to several proposed models, the dynamic equilibrium between SecA monomers and dimers is important for protein translation. Recently, several single-molecule analyses have been performed to identify the functional unit of Sec complex [22]; however, a consensus model of protein translocation has not been proposed to date. Further investigation of the Sec machinery requires an experimental system in which the oligomeric states of SecA and SecYEG are controlled. In order to measure protein translocation using various experimental devices, especially for single-molecule studies, a highly purified and simple single-unit system is necessary. In this study, we evaluated the potential of a fusion protein of SecY and SecA connected by a linker, to serve as a simplified experimental tool for studying protein translocation.

## Materials and methods

### Purification of SecYAEG complex

The genes encoding SecY(L2V, R252G)–(GGSG)_4_–SecA_1-939_–His_10_ fusion protein and SecE and SecG from *Thermus thermophilus* HB8 [5] were inserted into the NcoI–XbaI, XbaI-HindIII, and HindIII sites in the pTV118N vector (Takara), respectively. The resultant plasmid and pAK22 [5] were introduced into *Escherichia coli* strain BL21 (DE3) cells (BioLabs Inc.). Cells were cultivated at 37°C to an A_600_ of approximately 0.7 in LB medium, supplemented with 50 μg/ml ampicillin and 20 μg/ml chloramphenicol. The expression of SecY-A, SecE, and SecG were then induced with 1 mM isopropyl β-D-1-thiogalactopyranoiside at 25°C for 16 h. Total membrane fraction was prepared as described previously [23] and solubilized in 20 mM Tris-HCl (pH 8.0), 300 mM NaCl, 5% glycerol, 20 mM imidazole, 2% *n*-Dodecyl-β-D-maltoside (DDM), and 0.1 mM 4-(2-aminoethyl) benzenesulfonylfluoride hydrochloride (Pefabloc). After ultracentrifugation (138,000 × *g*, 60 min, 4°C), the supernatant was mixed with Ni-NTA Agarose (QIAGEN) equilibrated with buffer A (20 mM Tris-HCl [pH 8.0], 300 mM NaCl, 5% glycerol, 1 mM Pefabloc, and 0.1% DDM), containing 20 mM imidazole, for 30 min at 4°C. The resin was washed with buffer A containing 40–60 mM imidazole. Then, SecYAEG complex was eluted with buffer A containing 300 mM imidazole. In order to lower the concentration of NaCl, the elution was repeatedly concentrated and diluted with 20 mM Tris-HCl (pH 8.0), 50 mM NaCl, 5% glycerol, and 0.1% DDM using an Amicon Ultra filter (Ultra-15, MWCO 100 kDa, Merck Millipore) and then loaded on a Hitrap SP HP column (GE Healthcare). After washing with the same buffer, SecYAEG was eluted with a linear gradient of elution buffer (50–1,000 mM NaCl, 20 mM Tris-HCl (pH 8.0), 5% glycerol and 0.1% DDM). For further purification, the SecYAEG-containing fraction was concentrated using the Amicon Ultra filter and loaded on a Superose 6 Increase 10/300 GL column (GE Healthcare) equilibrated with buffer A.

### Preparation of preproteins

Preproteins *E*. *coli* proOmpA and proOmpA mutant (L59) with His and Myc tags at the C-terminus were prepared as described previously [24]. The proOmpA(L59) mutant forms an intramolecular 59-amino acid loop (L59) caused by the introduced disulfide bond, which sterically hampers its translocation. An inactive mutation (residues 8–10 IVA to QQQ) in the signal peptide of proOmpA, called 3Q [6], was introduced by site-directed mutagenesis. The proOmpA(L59-3Q) mutant was purified in the same manner [24]. For preparing the proOmpA(L59)-sfGFP (superfolder GFP [25]), a C-terminal-extended proOmpA mutant, we constructed a plasmid expressing proOmpA(L59)_1-352_–TS–sfGFP–TS–H_6_. Expression in *E*. *coli* cells of *secY24* mutant was induced as described previously [24]. Cells were suspended in 50 mM Tris-HCl (pH 8.0), 1 M NaCl, 8 M Urea and disrupted by sonication. After centrifugation (20,000 × *g*, 15 min, 20°C) and ultracentrifugation (138,000 × *g*, 30 min, room temperature), the supernatant was mixed with Ni-NTA Agarose (QIAGEN) equilibrated with 50 mM Tris-HCl (pH 8.0), 1 M NaCl, 8 M urea, and 20 mM imidazole for 30 min. The resin was washed with the equilibration buffer. Then, proOmpA(L59)-sfGFP was eluted with 50 mM Tris-HCl (pH 8.0), 1 M NaCl, 8 M urea, and 300 mM imidazole. The concentration of NaCl in the eluate was diluted to 20 mM with 50 mM Tris-HCl (pH 8.0) and 8 M urea. The solution was loaded on a Hitrap Q HP column (GE Healthcare) equilibrated with 50 mM Tris-HCl (pH 8.0) and 8 M urea. After washing with the same buffer, proOmpA(L59)-sfGFP was eluted with a gradient of elution buffer (50–250 mM NaCl, 50 mM Tris-HCl [pH 8.0] and 8 M urea).

### Preparation of liposomes and *in vitro* protein translocation assay

SecYEG- and SecYAEG-reconstituted proteoliposomes were prepared as described previously [26]. Protein translocation was initiated by adding proOmpA (final concentration of 0.02 mg/ml) to a mixture containing 50 mM Tris-HCl (pH 8.0), 0.5 mg/ml BSA, 5 mM MgSO_4_, 0 or 5 mM ATP, 5 mM DTT, 0.3 μM SecYEG or SecYAEG in liposomes, and 0 or 0.3 μM SecA_1-939_, and incubated at 50°C for 20 min. The samples were then treated with proteinase K (0.1 mg/ml) for 20 min on ice. After SDS-PAGE, proOmpA was detected by anti-Myc immunoblotting [26].

In order to detect the protein translocation intermediate, the protein translocation reaction was performed using proOmpA(L59). The reaction was performed in the same manner as that with proOmpA, except for the presence or absence of a reductant. After SDS-PAGE, proOmpA was detected by anti-proOmpA_38-54_ immunoblotting.

### Interaction between sec-reconstituted nanodiscs and preprotein

The protein-lipid mixture containing 10.8 mg/ml SecYAEG complex, 7.8 mg/ml MSP1D1, a membrane scaffold protein [27], 8 mg/ml *E*. *coli* phospholipids (Avanti) in 20 mM Tris-HCl (pH 8.0), 300 mM NaCl, 5% glycerol, 0.1% DDM, and 0.1 mM Pefabloc was gently mixed at 4°C for 1 h. Bio-Beads SM2 (Bio-Rad) were added to the mixture to remove the detergent. After gently mixing at 4°C overnight, the mixture was filtrated using Centrifugal Filters PVDF 0.22 μm (Millipore), or by ultracentrifugation (109,000 *g* × 30 min, 4°C), and then loaded on a Superdex 200 10/300 GL column (GE Healthcare) equilibrated with 50 mM HEPES-NaOH (pH 8.0) and 300 mM NaCl. The fractions containing the SecYAEG-reconstituted nanodisc were pooled.

The nanodiscs and preproteins were mixed with 50 mM HEPES-NaOH (pH 8.0), 0 or 5 mM MgSO_4_, 0 or 5 mM ATP, 300 mM NaCl and incubated at 37°C for 30 min. The samples were mixed with native PAGE sample buffer containing 50 mM Tris-HCl (pH 8.0), 5 mM MgSO_4_, 0 or 5 mM ATP, and 20% glycerol. After native PAGE using SuperSep Ace (Wako), the proteins were dyed with Coomassie Brilliant Blue.

## Results

### Protein translocation activity of SecY-SecA fusion protein

**Fig 1:**
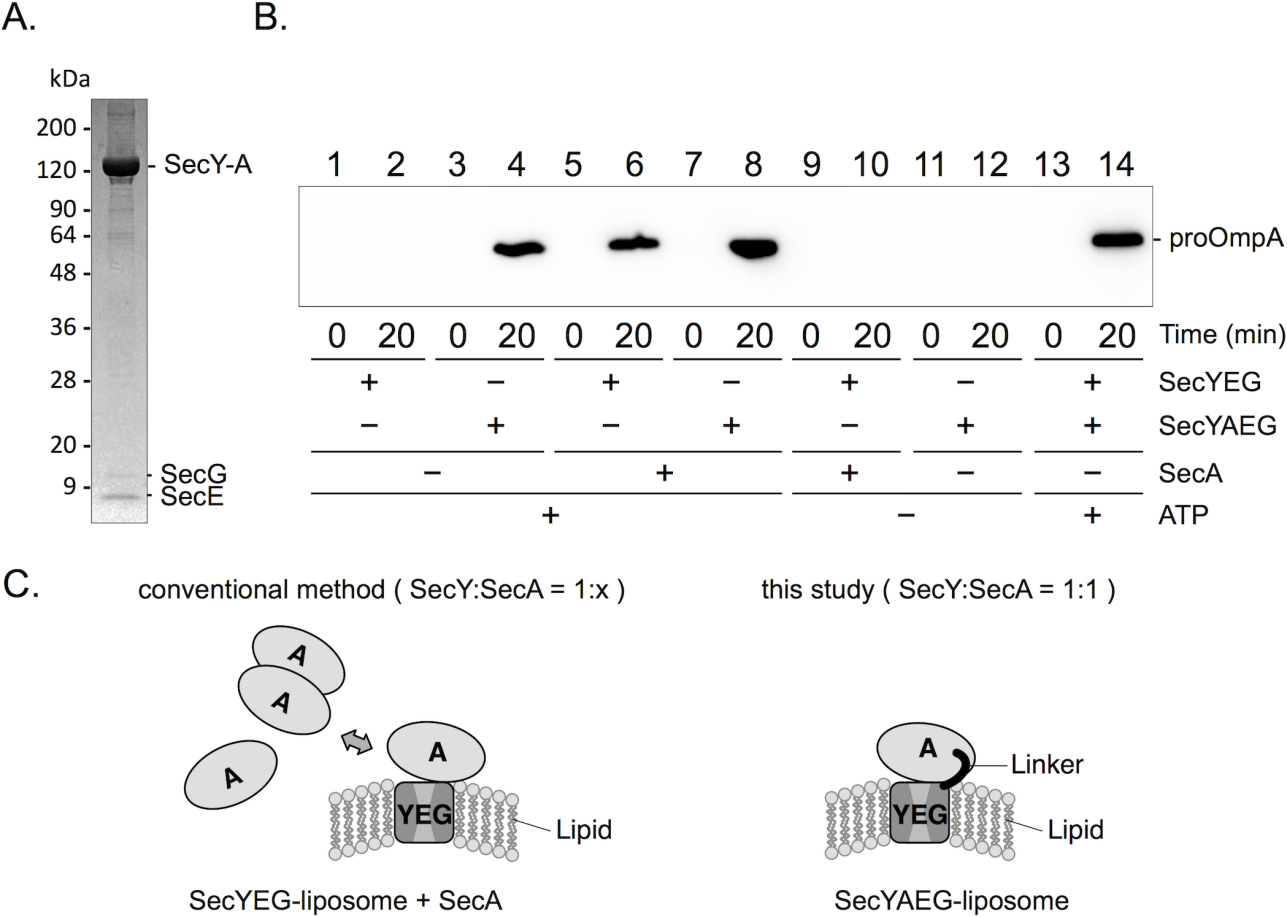
Protein translocation activity of the SecYAEG complex embedded in liposomes. A, Purified SecYAEG complex analyzed by SDS-PAGE and Coomassie Brilliant Blue staining; B, Protein translocation assay using SecYAEG-reconstituted or SecYEG-reconstituted liposomes in the presence or absence of SecA ATPase; protease-resistant translocated substrate, proOmpA-His_6_-Myc, was detected by anti-Myc immunoblotting. C, Schematic depiction of the protein translocation systems *in vitro*.

In the present work, we prepared the construction of the SecY-SecA fusion protein, with a linker (GGSG)_4_ connecting the C-terminus of SecY and the N-terminus of SecA. The C-terminal disordered region of SecA (~50 amino acid residues) was removed. For purification, the His_10_-tag was attached to the C-terminus. The SecY-A/SecE/SecG complex was purified by 3-step column chromatography (Fig 1A). The SecYAEG-reconstituted liposomes drove the translocation of proOmpA, a substrate protein, across the membrane after addition of ATP (Fig 1B lanes 3 and 4, and 1C). The translocation activity of SecYAEG-reconstituted liposomes seems to be similar to SecYEG-reconstituted liposomes (Fig 1B lanes 4 and 6, and 1C). Remarkably, the activity of the SecYAEG-reconstituted liposomes did not change significantly in the presence of additional SecYEG or SecA molecules (lanes 7,8,13,14), implying that SecYAEG-reconstituted liposomes are sufficient to mediate protein translocation activity *in vitro*. Considering the fact that SecYEG functions as a monomer in SecA-driven protein translocation [6, 28], one unit of SecYAEG may be the minimum constitution required for protein translocation.

### One unit of SecYAEG complex interacts with preprotein

**Fig 2:**
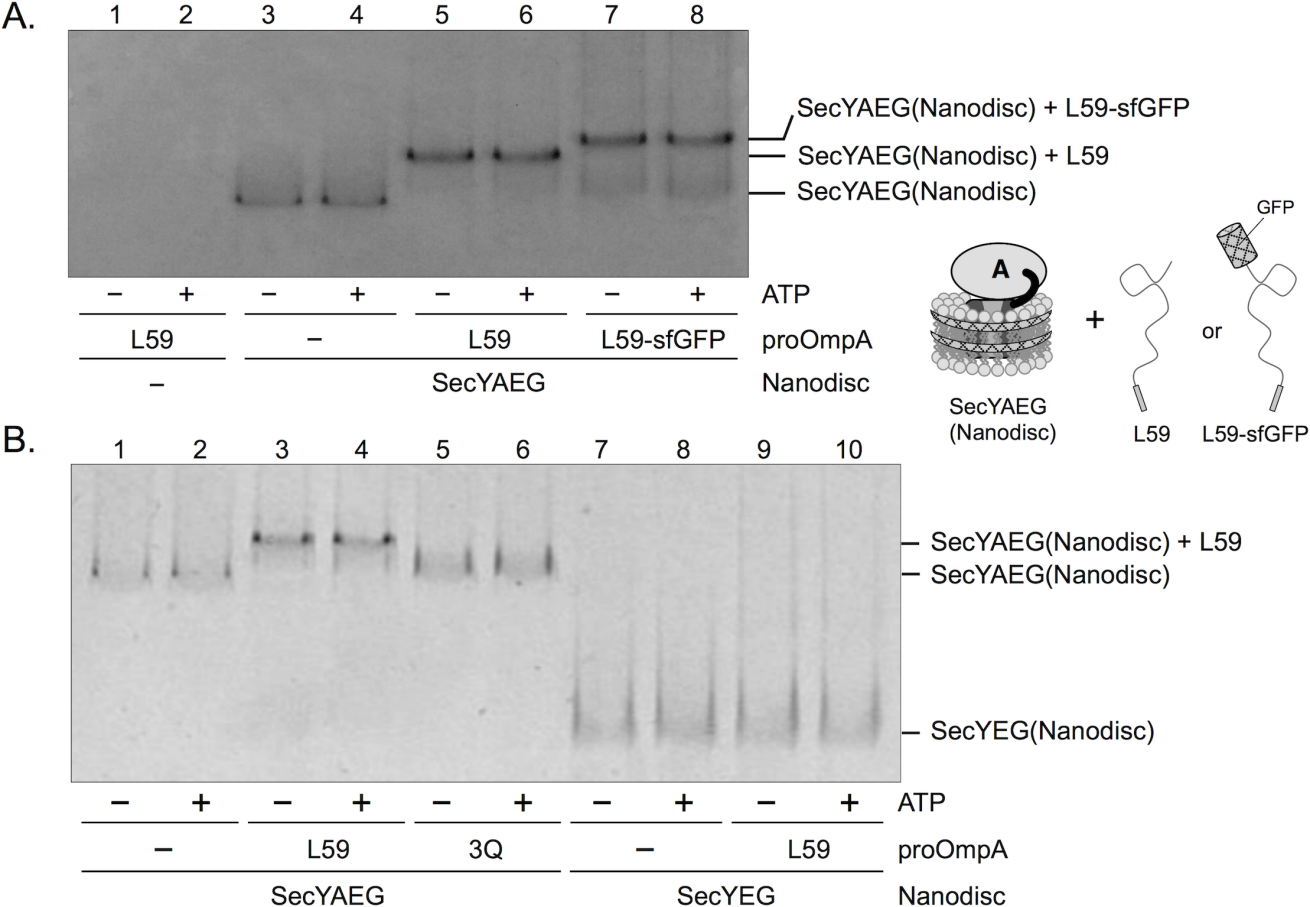
Stable complex of SecYAEG-reconstituted nanodisc and proOmpA. A, The mixture of SecYAEG-reconstituted nanodisc and proOmpA(L59) or proOmpA(L59)-sfGFP mutants, containing a disulfide loop nontranslocatable though the SecYEG, was analyzed by native PAGE and Coomassie Brilliant Blue staining. These bands were interpreted as stable complexes of SecYAEG-reconstituted nanodiscs with or without proOmpA. The upshifted bands were unaffected by the presence or absence of ATP. Schematic diagrams of the prepared proteins are shown. B, The upshifted bands were not detected using a signal peptide mutant proOmpA, 3Q, or SecYEG-reconstituted nanodisc instead of SecYAEG-reconstituted nanodisc.

Previous fluorescence resonance energy transfer studies have revealed that, during protein translocation, an intermediate complex is formed in the system from SecYEG-reconstituted nanodiscs, SecA, and a preprotein [28]. We prepared SecYAEG embedded in nanodiscs in a similar manner as previously reported [29]. Native PAGE data showed that the SecYAEG-reconstituted nanodiscs migrated more slowly than the SecYEG-reconstituted nanodiscs (Fig 2B lanes 1, 7). Here, we investigated whether the SecYAEG-reconstituted nanodiscs recognize proOmpA. The proOmpA(L59) mutant possesses an intramolecular 59-amino acid loop, which is formed as a result of introduced double cysteine residues [24], inducing the intermediate of protein translocation due to steric hindrance from the loop. Using this mutant as a substrate in protein translocation, most of the bands of the SecYAEG-reconstituted nanodiscs were found to be slightly upshifted. (Fig 2A, lanes 5, 6 and 2B lanes 3, 4). However, the upshifted bands were not detected in the presence of a signal peptide mutant of proOmpA(L59), called 3Q, which inhibited the interaction between the signal peptide and Sec proteins [6] (Fig 2B lanes 5,6). When the proOmpA(L59) mutant bound to superfolder GFP at the C-terminus (L59-sfGFP) [25], which is approximately 27 kDa larger than proOmpA(L59), was used, the upshifted bands migrated at a slightly slower rate in the native PAGE (Fig 2A lanes 7, 8). These data strongly indicated that the upshifted bands comprised the SecYAEG-reconstituted nanodiscs complexed with the preprotein. In addition, the bands of SecYEG-reconstituted nanodiscs remained unchanged in the presence of proOmpA (Fig 2B lanes 9, 10), implying that SecA is required for the binding of the precursor to the SecYEG embedded in the lipid bilayer. The upshifted bands appeared in the presence and absence of ATP, suggesting that ATP is not necessary for the recognition of signal peptides and the formation of the protein translocation complex comprising SecYAEG-reconstituted nanodiscs and proOmpA. The fusion SecY-SecA protein enables the utilization of SecYAEG-reconstituted nanodiscs as a simplified tool for single-molecule analyses of protein translocation.

### Protein translocation intermediates generated by the SecYAEG complex

**Fig 3:**
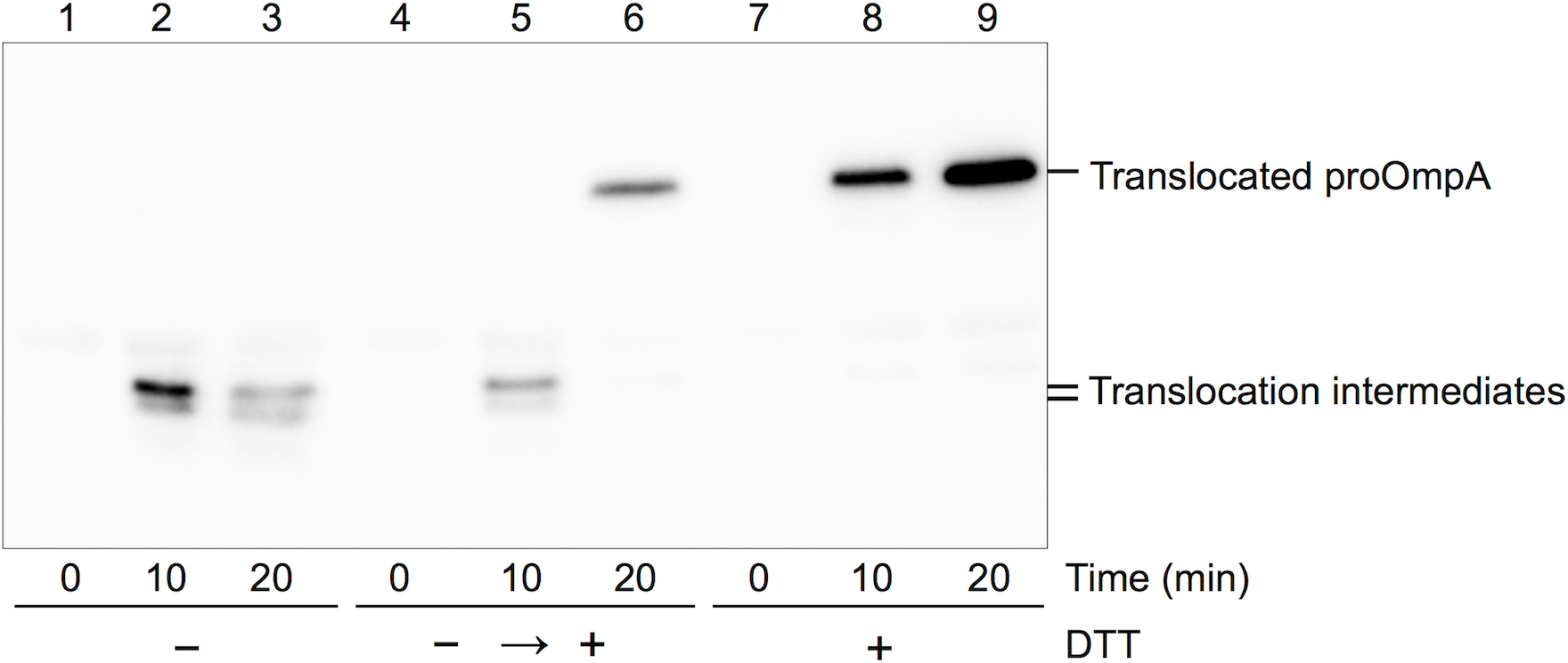
Detection of protein translocation intermediate derived from SecYAEG-reconstituted liposomes and proOmpA(L59) mutant. The intermediate states were generated using proOmpA(L59) mutant. The protease-resistant region of proOmpA in the intermediate states was detected by newly obtained anti-proOmpA_38-54_ immunoblotting under non-reducing conditions. Translocation was re-initiated upon addition of DTT, a reductant.

In order to observe protein translocation mediated by a single unit of Sec machinery, the ability to prepare translocation intermediates using SecYAEG-reconstituted nanodiscs is necessary. When proOmpA(L59) is used as a substrate, protein translocation is halted at the intermediate state due to the internal disulfide loop of proOmpA. After addition of DTT, a reductant, the protein translocation reaction is re-initiated by the intermediates (Fig 3 lanes 4–6). In this study, we prepared a rabbit polyclonal antibody against the subsequent sequence (residues 38–54) of proOmpA signal peptide. The antigenic region is translocated across the membrane at an early stage. Although we were previously unable to detect the protein translocation intermediates using antibodies against commonly used C-terminal tags, the newly prepared antibody could detect the two bands of the intermediate formed by SecYAEG-reconstituted liposomes, after immunoblotting without using isotopes (Fig 3 lanes 2, 3 and 5). The data suggest that there are at least two stable intermediate states with slightly different conformations. The addition of DTT further enabled observation of the latter step of protein translation, from the intermediate state to completion. The present simplified experimental system using the fusion protein SecY-SecA should be useful for studying the protein translocation process. An additional advantage of the present system is that the antibody prepared in this study enables the detection of intermediates without requiring the use of isotopes.

## Discussion

In order to perform the protein translocation assay *in vitro*, it is necessary to mix at least three proteins, namely SecA ATPase, proOmpA, and membrane-embedded SecYEG, before the incubation for the protein translocation reaction. Each SecYEG complex reconstituted within liposomes exists randomly as one of two different topology states. Further, it is not possible to determine whether each SecYEG complex, even when present in the correct topology, is active. Therefore, it is particularly difficult to accurately determine the number of active SecYEG complexes reconstructed within liposomes with the correct topology, and consequently not possible to accurately adjust the stoichiometry of the active SecYEG and SecA. The present standard procedure including the protein mixing step results in excess or deficiency of SecA or SecYEG molecules. Even if the stoichiometry is matched, SecYEG and SecA do not continuously form a stable SecYEG-SecA complex, resulting in the sample becoming heterogeneous. Therefore, the use of procedures that involve mixing Sec proteins during sample preparation should be avoided. Although the oligomer states of SecYEG and SecA are still controversial, as described in the Introduction, we were able to completely fix the stoichiometry of SecYEG and SecA using the fusion protein SecY-SecA, without the requirement for complex procedures. We showed that one unit of SecYAEG binds tightly to the substrate, and that SecYAEG exhibits protein translocation activity and forms stable intermediates. The addition of excess SecA and SecYEG did not significantly elevate or decrease this activity. These results support the idea that SecYAEG is sufficient for mediating protein translocation. Although we cannot exclude the validity of working models with multiple units, the present experimental system using SecYAEG-reconstituted nanodiscs should be useful for single-molecule analyses of protein translocation. For example, atomic force microscopy and single-molecule fluorescence resonance energy transfer allow us to observe and visualize protein translocation mediated by the SecYAEG complex in real time. Therefore, the present fusion protein should be suitable for various additional studies, including quantitative biophysical approaches. The present system should enable detailed analyses of the Sec translocon machinery. Protein translocation requires SecYEG, SecA ATPase, appropriate membrane structure, and the substrate protein, which together constitute a considerably complex system. The simplification of sample should enable the study of the Sec protein translocation system in more detail.
